# Genetics of destemming in pepper: A step towards mechanical harvesting

**DOI:** 10.3389/fgene.2023.1114832

**Published:** 2023-03-17

**Authors:** Theresa Hill, Vincenzo Cassibba, Israel Joukhadar, Bradley Tonnessen, Charles Havlik, Franchesca Ortega, Sirisupa Sripolcharoen, Bernard Jurriaan Visser, Kevin Stoffel, Paradee Thammapichai, Armando Garcia-Llanos, Shiyu Chen, Amanda Hulse-Kemp, Stephanie Walker, Allen Van Deynze

**Affiliations:** ^1^ Seed Biotechnology Center, University of California, Davis, Davis, CA, United States; ^2^ Department of Extension Plant Sciences, New Mexico State University, Las Cruces, NM, United States; ^3^ Los Lunas Agricultural Science Center, Los Lunas, NM, United States; ^4^ Department of Plant and Environmental Sciences, New Mexico State University, Las Cruces, NM, United States

**Keywords:** *Capsicum annuum*, pepper, fruit abscission, mechanical harvest, green fruit harvest, paper pot, destemming

## Abstract

**Introduction:** The majority of peppers in the US for fresh market and processing are handpicked, and harvesting can account for 20–50% of production costs. Innovation in mechanical harvesting would increase availability; lower the costs of local, healthy vegetable products; and perhaps improve food safety and expand markets. Most processed peppers require removal of pedicels (stem and calyx) from the fruit, but lack of an efficient mechanical process for this operation has hindered adoption of mechanical harvest. In this paper, we present characterization and advancements in breeding green chile peppers for mechanical harvesting. Specifically, we describe inheritance and expression of an easy-destemming trait derived from the landrace UCD-14 that facilitates machine harvest of green chiles.

**Methods:** A torque gauge was used for measuring bending forces similar to those of a harvester and applied to two biparental populations segregating for destemming force and rate. Genotyping by sequencing was used to generate genetic maps for quantitative trait locus (QTL) analyses.

**Results:** A major destemming QTL was found on chromosome 10 across populations and environments. Eight additional population and/or environment-specific QTL were also identified. Chromosome 10 QTL markers were used to help introgress the destemming trait into jalapeño-type peppers. Low destemming force lines combined with improvements in transplant production enabled mechanical harvest of destemmed fruit at a rate of 41% versus 2% with a commercial jalapeńo hybrid. Staining for the presence of lignin at the pedicel/fruit boundary indicated the presence of an abscission zone and homologs of genes known to affect organ abscission were found under several QTL, suggesting that the easy-destemming trait may be due to the presence and activation of a pedicel/fruit abscission zone.

**Conclusion:** Presented here are tools to measure the easy-destemming trait, its physiological basis, possible molecular pathways, and expression of the trait in various genetic backgrounds. Mechanical harvest of destemmed mature green chile fruits was achieved by combining easy-destemming with transplant management.

## 1 Introduction

Cultivated *Capsicum spp*., include *C. annuum, C. frutescens, C, chinense, C. buccatum* and *C. pubescens,* with *C. annuum* the most used by far in the United States. They are grown for vegetable, spice, ornamental, medicinal and lachrymator uses and are a rich source of vitamins A, B, and C, and consumed to improve diets around the world (Howard et al., 2000; Bosland et al., 2012; Ganguly et al., 2017; Saleh et al., 2018). They are also high in iron, potassium and magnesium (Pawar et al., 2011). Peppers drove a $1.4 B salsa industry and $1.6 B hot sauce market in the United States in 2020 with increasing demand (IBISWORLD, 2021). The per acre production costs for bell peppers is estimated to be $13,235 for fresh market and $6,650 for processing in United States. For smaller-fruited specialty peppers such as jalapeños and other chiles, estimated costs are similar to or even greater than bells. The majority of peppers in the United States for fresh market and processing are handpicked, and harvesting can account for 20–50% of production costs (Takele et al., 2013). In addition, processed peppers command significantly lower prices for growers, yet have the same high labor costs as fresh market. Consequently, mechanical harvesting has been identified as a key goal for the pepper industry (Funk and Walker, 2010).

Mechanical harvesting of peppers is not novel with attempts as early as the 1960s by adapting tomato harvesters (Marshall, 1995; Marshall and Boese, 1998; Shooter and Buffinton, 1999; Walker and Funk, 2014). Germplasm development has resulted in peppers that detach well from the stem. *Detachment* is separation of pedicel from the stem and *destemming* (decapping) is separation of the fruit from the calyx and pedicel. Efforts have been made to optimize fruit detachment for mechanical harvesters by some breeding programs. For example, advancements have been made in varieties such as the paprika-type NuMex Garnet, evaluated for mechanical harvest traits of red ripe fruits by New Mexico State University (NMSU) (Walker et al., 2004). These efforts contributed to widespread adoption of mechanical harvesting of red chiles in NM (Walker and Funk, 2014). However, green peppers such as New Mexico-type green chiles, jalapeños, serranos, etc., continue to be largely hand-harvested (Walker et al., 2021). This has resulted in the continued loss of green chile production from the US to countries with much lower labor costs. Destemming, along with fruit damage, are intransigent challenges that have prevented mechanical harvest adoption in New Mexico-type green chile (Walker and Funk, 2014). Green chiles pose particular challenges to mechanization, as more force is needed to detach the pedicel from the stem than ripe (red) fruits. Additionally, the calyx and pedicel must be removed from the fruit (destemmed) for processing; this extraneous material presents quality and safety issues in processed chile peppers (Herbon et al., 2009; Funk et al., 2011). Compared to red fruit used for drying, damage of green fruit is not tolerated in the market.

A systems approach is required to enable mechanical harvesting that includes developing pepper varieties that are amenable to mechanical harvest, combining traits for easy picking, destemming, plant architecture, uniform maturity and low fruit breakage (Palevitch and Levy, 1984; Marshall, 1997). This needs to be combined with crop management and harvester mechanism (Funk and Walker, 2010; Funk et al., 2011). To address this effort, we describe an important component of this goal that enables destemming of fruit from the pedicel while maintaining fruit quality when harvested mechanically.

## 2 Materials and methods

### 2.1 Plant material and experimental sites

Crosses were performed between an easy-destemming serrano-type Mexican landrace UCD-14 and the non-destemming cultivars “Maor”, a blocky-type, and “Garnet”, a paprika-type (Chaim et al., 2001; Zhang et al., 2004). The resulting F_1_ plants were self-pollinated in the greenhouse to generate the MUC14 (Maor × UCD-14) and GUC14 (NuMex Garnet × UCD-14) F_2_ populations, respectively. To generate F_3_ families, F_2_ individuals were selfed by open-pollination in in field plots at the Plant Sciences field station at the University of California, Davis (UC Davis) in 2014 and 2015. In April of 2015, MUC14 and GUC14 F_3_ families were planted and grown in the greenhouse to the six-leaf stage, transferred to a lathhouse for 1 week, then transplanted by hand into field plots in a Reiff series silt loam soil (coarse-loamy, mixed, superactive, nonacid, thermic Mollic Xerofluvents) at UC Davis. Field plots were on 60-inch beds with double rows spaced at 14 inches off center and drip tape at 8 inches below each row. Selections were advanced to F_4_ to F_7_ by self pollinating in the field or greenhouse. In most cases, field plants were covered with 3 × 4 ft pollination bags (Vilutis and Co., Inc., Frankfort, Illinois, United States) to ensure self pollination.

### 2.2 Phenotyping for destemming

Destemming rate and force were measured using a Mark-10 M4-200 Digital Force Gauge (Copiague, NY) with a wire mesh Kellems grip (Hubbell Incorporated, Shelton, CT, United States) used to grip the fruit in 2015. In subsequent years, destemming was measured using a Tohnichi Torque Gauge BTG90CN-S (Tohnichi America Corporation, Buffalo Grove, IL, United States). The destem rate was the fraction of fruits that the pedicel with calyx was removed cleanly from the fruit without breakage of the fruit or pedicel/calyx. A total of 15–30 fruits/plot were assayed to determine destemming rate. Destemming force was the mean force to remove the stem among the fraction of fruits destemmed for each plot.

For quantitative trait trait loci (QTL) mapping and the selection of lines to advance, F_3_ families were evaluated in 2015–2019 (Supplementary Data Sheets S1, S2). In 2015, 112 GUC14 and 38 MUC14 F_3_ families were phenotyped (Supplementary Data Sheet S3). An additional 251 MUC14 families were grown in 2016. The F_3_ families grown in 2016 were screened by hand for destemming rate, gripping the fruit and using the thumb to apply force and remove the pedicel and calyx at a minimum of five fruits per plot. A total of 46 lines had a frequency of destemming by thumb equal to or greater than one in five and were evaluated further using the torque gauge. A In 2019, a random set of 151 MUC14 F_3_ families from the previously evaluated 289 were evaluated for destemming force and frequency using the torque gauge. A randomized complete block design (RCBD) with two replications of six plants were scored in 2015 and 2016 and three replications of six plants in 2019. A total of 15 fruits from five plants per plot were measured for destemming rate and force. In 2015 and 2016, fruit size and shape data was also collected as described in Chunthawodtiporn et al. (2018).

Based on both high destemming rate and low destemming force, F_3_ families were selected to advance. Evaluation of selections was carried out in 2016, 2017, 2019, and 2020. The UCD-14 parent was also included in each trial. An RCBD was employed with three replications of 10 plants per replicate with 30 fruits phenotyped per plot. In 2016, selected families were assayed for both pull and torque destemming rate and force. The 2017 thru 2020 evaluations were done with the torque gauge. In 2016, a total of 11 MUC14 and four GUC14 F_3_ families plus three MUC14 F_4_ families were evaluated. In 2017, a total of five F_3_ and 14 F_4_ MUC14 F_3_ families along with four commercial jalapeño hybrids were evaluated. The 2019 trial consisted of six commercial jalapeño hybrids and 29 F_4_ to F_6_ families representing 12 MUC14 F_2_. The 2020 trial consisted of four commercial jalapeño hybrids and 46 F_4_ to F_7_ families derived from 18 MUC14 F_3_ families.

### 2.3 Mechanical harvest trials

MUC14 selections MUC14-200, MUC14-17 and MCU14-330 were included in a replicated mechanical harvest field trial in 2018 at NMSU’s Los Lunas Agricultural Science Center as previously described (Walker et al., 2021). The trial was arranged in a RCBD with the MUC14 entries having two replications. The field was direct-seeded then thinned to single plants spaced eight inches apart. Row spacing was 30 inches and the field was furrow irrigated and fertilized according to standard local practices (Bosland and Walker, 2014). New Mexico–type green chile pepper cultivars (Havlik et al., 2018) were grown as standard controls for harvest of fruits with stems.

Cultural practices for mechanical harvest trials at UC Davis were refined in 2019, 2020, and 2021. Transplants were produced as described above. Seedlings were transplanted by hand after 5 weeks in the greenhouse followed by 1 week in the lath house. Row spacing was 30 inches with drip tape buried at eight inches and plant spacing at one foot within rows. Plots were 34 ft long and end plants were removed before harvest. A carousel planter set for one foot spacing was used in 2021. In 2019, six MUC14 easy-destemming selections MUC14-37, -179, -191, -200, -228, and -297 were included. In 2020, 14 entries were included (four jalapeño commercial hybrids, three New Mexico-type chiles and seven selections: MUC14-37, -89, -139 (2 seedlots), -200, -228, -297). In 2021, 16 entries (two commercial jalapeño hybrids, four New Mexico-type chiles, two MUC14 selections -139 and -228, and eight jalapeño x MUC14 BC_1_S_3_ selections) were included. All UC Davis trials were arranged in a RCBD with four replications. The 2020 trial also consisted of two locations that differed by soil type, Reiff very fine sandy loam and Yolo silt loam. In 2022, entries were seeded into 128 cell non-chain paper pots (Small Farm Works, Reeseville, United States). After 3 weeks in the greenhouse, seedlings were moved to the lath house for 1 week and then transplanted by hand.

In each trial, harvest was scheduled for peak season when most fruit were fully sized and marketable for green chile peppers (horticulturally mature) and not predominately red (physiologically mature). Immediately before harvest, destemming force was measured in the same experimental plots used for mechanical harvest trials. Plots were harvested with a one-row, tractor powered Etgar Series MOSES 1010 (Etgar LTD., Bet- Lehem-Haglilit, Israel) mechanical harvester equipped with a picking head equivalent to the commercial Yung-Etgar evaluated in earlier field tests (Funk and Walker, 2010). The machine proceeded through the field at 1.1 mph; the counter-rotating helix head speed was adjusted as needed for even flow of fruit on the conveyors at the start of the trial. All material picked from each plot was bagged separately. All fruit left attached to the plants and on the ground in each plot following the harvest was collected. Fresh fruit weights obtained from these two categories represented unharvested fruit. The harvested material was promptly transferred to a plant processing laboratory and sorted into categories: destemmed marketable green fruit, marketable green fruit with pedicel attached, marketable green fruit attached to branches, broken or damaged fruit, red fruit, and diseased fruit. The fresh weight was obtained for each category of sorted material for each plot.

### 2.4 SNP detection using genotyping-by-sequencing

DNA was extracted from F_3_ seed using a previously published CTAB extraction method (Xin et al., 2012), with an additional clean-up step with Agencourt AMPure XP Bead protocol #E6260 (Beckman Coulter, Brea, CA, United States). DNA concentration was quantified using a picogreen assay and/or Qubit 2.0 Fluorometer. Sequencing libraries were constructed with a modified protocol that produces libraries with an average size of 300–350 bp (Monson-Miller et al., 2012). Modifications included using the blunt-end cutting restriction enzyme MlyI for enzymatic shearing and optimization of temperature and incubation periods for pepper. Genomic libraries were delivered to the DNA Technologies and Expression Analysis Core at the UC Davis Genome Center for sequencing, which included the parental lines UCD-14, “Garnet,” and “Maor.” The parental libraries were used to determine the parental alleles. The samples were sequenced on an Illumina HiSeq 4000, which produces 100–150 bp paired end reads.

Raw sequence data were trimmed to remove the Illumina adapter sequences, and soft clipped based on sequencing quality with Trimmomatic with default parameters for paired-end reads (Bolger et al., 2014). Adapter-trimmed and quality-clipped reads were then mapped to the latest pepper reference genome UCD v1.0 (Hulse-Kemp et al., 2018) with Bowtie2 on the sensitive setting (Langmead et al., 2012). Only the uniquely mapped reads were used as input for Samtools (version 0.1.7a) to extract all of the genetic variants. These sequences were then filtered with an in-house filtering pipeline (Ashrafi et al., 2012). Read variants with phred-scaled quality score lower than 20, sequencing coverage higher than 200 and lower than 4, and consensus sequence quality lower than 20 were filtered out. Read variants with the major allele frequency equal to or higher than 0.9 were set to homozygous.

### 2.5 Genetic mapping

To generate genetic maps for each population the following filters were applied: taxa maximum of 70% missing data, SNPs maximum of 10% missing data, SNP allele frequencies between 0.2—0.8 allowed and SNP heterozygous calls 0.2—0.7 allowed. After removing taxa with high missing data, MUC14 and GUC genetic maps were generated from 155 to 107 individuals, respectively. For each map, SNP markers with common haplotypes were collapsed into genomic bins based on likely recombination breakpoints calculated using the python script SNPbinner (https://github.com/solgenomics/SNPbinner, Gonda et al., 2019). The MUC14 genetic map was derived from 47,422 SNP markers collapsed into 1,485 genomic bins (Supplementary Data Sheets S4, S5). The GUC14 genetic map was from 20,466 SNP markers collapsed into 869 genomic bins (Supplementary Data Sheets S6, S7). Genetic maps were generated using MSTmap Online (http://mstmap.org/) (Wu et al., 2007; Wu et al., 2008).

### 2.6 Statistical analysis

Statistical analyses were carried out using JMP software (Cary, NC 27513-2414, United States). Outliers were detected using Cook’s D Influence >0.5 among samples and removed. Plot means then means and standard errors across replicates for each entry were calculated. Entry means comparisons were done using a one-way analysis of variance (ANOVA). Least significant difference (LSD) threshold matrix and Student’s t connecting letters reports were generated.

Quantitative trail trait loci analysis and heritability estimates were carried out in RStudio (RStudio Team, 2020). Heritability estimates were calculated from 151 MUC14 F_3_ families genotyped and phenotyped in 2019 using the R package sommer (Covarrubias-Pazaran, 2016; Covarrubias-Pazaran, 2018). Prior to QTL analysis, destemming force data were adjusted for spatial variation using the lmer function in the R-package:lme4, to calculate Best Linear Unbiased Predictions (BLUPs). QTL analyses were performed using composite interval mapping with QTL IciMapping (IciM) Version 4.2 (https://isbreedingen.caas.cn/software/qtllcimapping/294607.htm) (Meng et al., 2015) and multiple QTL modeling using the stepwiseqtl, makeqtl, and fitqtl functions in the R/qtl package (Broman et al., 2003). A Genome-wide association study was carried out in TASSEL and rMVP using pull force and frequency data collected in 2015 for the GUC14 and MUC14 populations combined (Supplementary Data Sheet S3). A total of 48,135 SNPs identified across both populations with a maximum of 10% missing calls were used and (Supplementary Data Sheet S8) (Bradbury et al., 2007; Yin et al., 2021) a mixed linear model (MLM) was used to account for population structure (Abecasis et al., 2000; Yu et al., 2006).

### 2.7 QTL validation

In 2019, individuals from families that were segregating for markers within QTL intervals on chromosomes 2, 3, and 10 were genotyped with markers CA.10X.chr2_143710406, CA.10X.chr3_245138565, and CA.10X.chr10_172967183; respectively (Supplementary Table S1). Genotyping was done using PCR Allele Competitive Extension (PACE) with PACE® 2.0 master mix and primer design by 3CR Bioscience (United Kingdom). Individuals were then grouped by genotype and transplanted to the field. All mature green fruits were phenotyped for destemming force with the torque gauge.

### 2.8 Phloroglucinol HCl stain

Flowers were tagged on the day of anthesis and harvested at 5–56 days after anthesis. After harvest, the fruit with pedicel were hand sectioned and subjected to phloroglucinol (Wiesner) stain for presence of lignin (Mitra and Loqué, 2014).

## 3 Results

### 3.1 Characterization of easy-destemming trait

**FIGURE 1:**
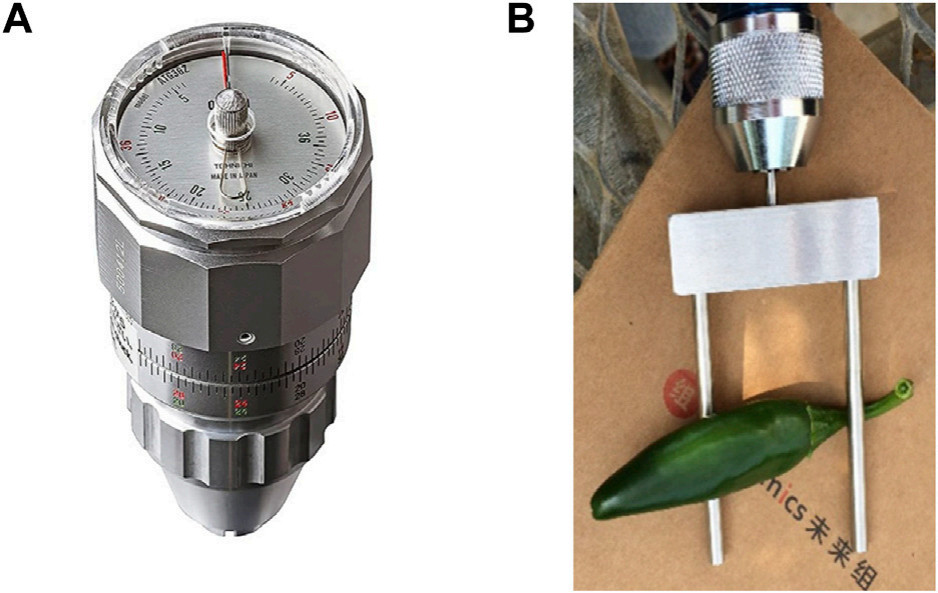
Torque Watch **(A)** with custom fork **(B)** to measure bending force of pepper fruit. This instrument measures the maximum bending force at breaking of pedicel from fruit when twisting.

A set of wild and semi-domesticated accessions was recently collected in regions of Mexico to identify potential sources of disease resistance and horticultural traits (Kraft et al., 2013). Among these is a semi-domesticated *C. annuum* accession, UCD-14, that has oblong fruit with firm texture, medium pericarp thickness and easily abscises when picked at the mature green stage, leaving the pedicel and calyx behind (destemmed). This line was crossed to a paprika-type chile pepper (“NuMex Garnet”) and a blocky type (“Maor”) to develop two F_2_ populations, GUC14 and MUC14, respectively. The easy-destemming phenotype was found segregating among the 38 MUC14 and 113 GUC14 F_2_ individuals in 2014 field trials at the Plant Sciences field station at UC Davis. Initial evaluation of green fruit destemming force was carried out in 2015 on MUC14 and GUC14 F_3_ families using a digital pull force gauge. For measurements in subsequent years, consistent with results from Miles et al. (1978), we developed a method to rapidly and reproducibly measure bending forces required for destemming using a Torque Gauge (Figure 1). The application of bending forces on the GUC14 F_3_ families resulted in a very high frequency fruit breakage at the pedicel attachment end and therefore the focus was concentrated on selecting MUC14 lines for advancement.

**FIGURE 2:**
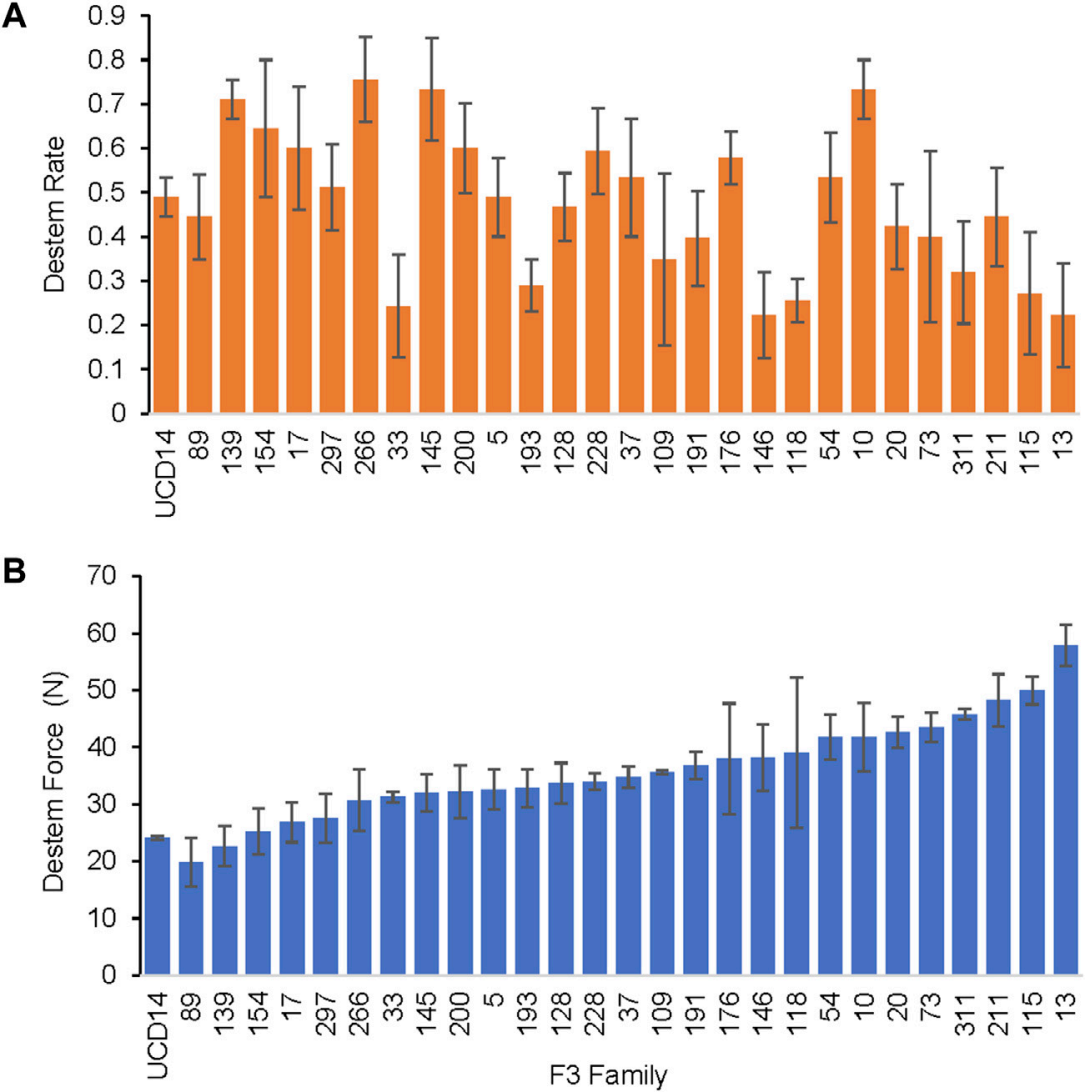
Trial in 2019 for QTL analysis. Representative MUC14 F_3_ families showing a range of destemming force and frequency with the torque gauge measured at the mature green stage. **(A)** Mean fraction of fruits cleanly destemmed and **(B)** mean destemming force for each family. Error bars represent standard error of replications.

A range of destemming frequency (fraction of fruit destemmed without breaking) and force was observed in MUC14 F_3_ families for both pull and torque measurements, with some families that had similar destemming force as UCD-14 at the mature green stage (Figure 2). The destemming trait was readily transferred across generations through phenotypic evaluation in the field at Davis from 2015 to 2020. Selections for low destemming force were made from 325 MUC14 F_3_ families evaluated in 2015 through 2017. A total of 35 F_3_ families were selected for further evaluation in replicated trials from 2016—2020 where a significant correlation of destemming force and frequency across years was observed (Supplementary Figure S1). A significant correlation was also found between pull and torque force assays (Supplementary Figure S1A). The F_3_ families used for QTL analysis that were evaluated in 2016 and 2019 showed significant but moderate correlation among trials, 0.57 for destem rate and 0.48 for destem force (Supplementary Figure S1B). Based on both destemming rate and force, 30 selected F_3_ families were advanced to F_4_, 10 to F_5_, four to F_6_ and two to F_7_. The 13 F_4_ to F_6_ families evaluated in 2019 and 2020 trials showed a moderate correlation for destemming rate (0.63) and a very high correlation of destem force (0.90) between years (Supplementary Figure S1C). The higher correlation with the more advanced lines may be due to fixation of the destemming trait or to a higher sampling rate, 90 total fruits for advanced selections verses 30 or 45 fruits for F_3_ families.

Fruit size and shape data were also collected for MUC14 F_3_ families. There were significant but moderate to low phenotypic correlations (0.32–0.53) between destemming force and fruit size and shape (Supplementary Figure S2). There was a significant negative but relatively weak correlation (0.231) between destem rate and pedicel end shape, where higher numbers reflected broader shoulders (IPGRI, 1995). The strongest correlations with destem force were observed for fruit width, pedicel end shape, and pericarp thickness.

### 3.2 Easy-destemming translates to machine harvestability

**FIGURE 3:**
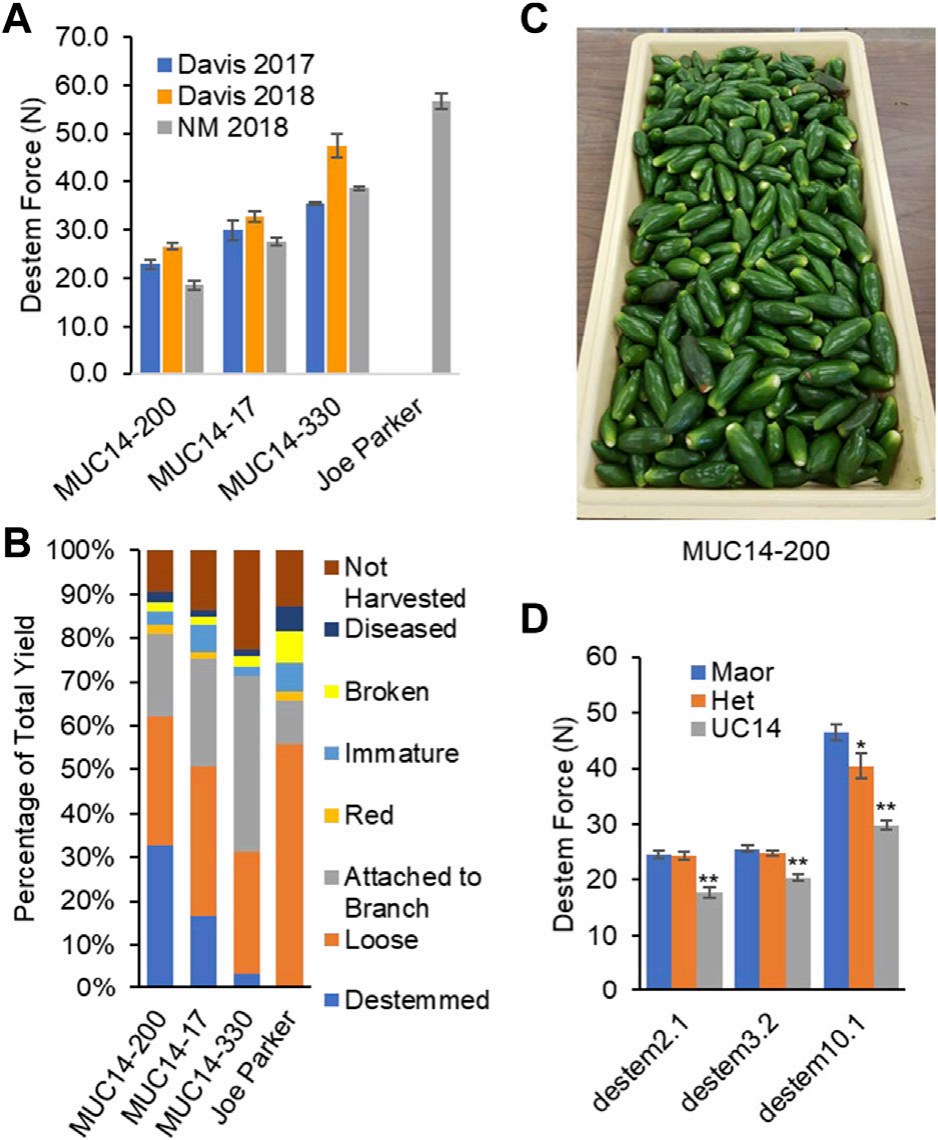
Machine harvest trial in 2018. **(A)** Lower destemming force (N) was correlated with the percentage of green destemmed fruits from an Etgar harvester. **(B)** Percent fruit classes: Destemmed is the mechanically harvested green marketable fruits perfectly destemmed, Loose is the mechanically harvested green marketable fruits with stem attached, and Attached to branch is the green marketable fruits born on harvested branches. Non-marketable fruits mechanically harvested are categorized into damaged, red, diseased and immature. Not harvested is the fruits remaining on plants or knocked to the ground and not recovered by the machine during harvest. **(C)** Fruits cleanly destemmed by the harvester. **(D)** Destemming force in newtons (N) among genotypes for families segregating at QTL. Plants homozygous for the UCD-14 allele had significantly lower destemming force (*p<0.05, **p<0.0001).

An initial replicated mechanical harvest trial was carried out in a replicated trial in NM in 2018. The UCD-14-derived selections performed well showing a direct correlation between destemming force and the proportion of peppers perfectly destemmed during mechanical harvest (clean break at receptacle/calyx without cracks in fruit, Figure 3). The majority of fruit were harvested with their pedicel attached. In 2018, only 0.8% of NuMex Joe E. Parker (a New Mexico-type variety selected for efficient mechanical removal of green fruit in previous tests) fruit harvested were destemmed during the mechanical harvest process, compared to the UC Davis easy-destemming line (MUC14-200) that had 32% destemmed fruit from mechanical harvest at mature green fruit stage (Figure 3).

The New Mexico-type varieties widely grown in NM are predominately open-pollinated and direct seeded. Most commercial jalapeños are hybrids where direct seeding isn’t feasible due to the higher cost of seeds. Standard transplants tend to differ in both root and shoot architecture when compared with direct-seeded plants, affecting mechanical harvest ability. Mechanical harvest of transplants was optimized at UC Davis during field trials from 2019 to 2022 (Table 1). Machine harvest trials in 2019 and 2020 at UC Davis were carried out using standard transplants and, in agreement with previous studies, resulted in plants lacking a strong root system and the above-ground vase shaped architecture with a minimal amount of basal branching that is required for efficient harvest with the Etgar harvester (Walker et al., 2021). An effort was made in 2020 to reduce the height and number of leaves of the transplants by adjusting greenhouse conditions. Application of water and fertilizer was every other day instead of every day and temperatures were reduce by 3°C after 3 weeks after seeding. This resulted in smaller, sturdier plants at transplant and a greater than 50% reduction in non-harvest fruits and two to three fold increase in harvested destemmed fruits (Table 1). In 2021, Flexxifloat 250 lifts (Flexxifinger, Assinoboia, Canada) were added to the Etgar harvester at UC Davis in an attempt to overcome challenges caused by the increased level of basal branching produced by transplants. The lifts were not effective in picking the plants from the ground. In 2022, transplants were grown in paper pots. This allowed transplantation after only 4 weeks, 2 weeks sooner than standard transplants. This resulted in plants with deeper roots and a more vase shaped architecture than observed in previous years (data not shown). Improvement in the proportion of harvested fruits and more than double the amount of harvested destemmed fruits was observed in 2022 versus improvements seen in 2020 (Table 1). The best performer in the 2018 NM trial, MUC14-200, had 42% of total yield (50% of harvested marketable yield) destemmed by the harvester in 2022.

**TABLE 1:** Summary of destemming rate and force from torque gauge along with mechanical harvest data collected at UC Davis in years 2019–2022 for MUC14 selections used for crosses to jalapeño along with a jalapeño commercial hybrid (Jal1). The 2022 trial also includes destemming introgressions into jalapeño for five—BC_1_S_3_ (20G722-) and one—F_4_ (Ds228MF76).

Line	Trial	Destem force (N)	Destem rate (%)	Mechanically harvested fruits				Not harvested^e^ (%)	Destemmed among harvested marketable
				Destemmed^a^	Loose^b^ (%)	Attached to branch^c^ (%)	Non-marketable^d^ (%)		
MUC14-200	2019	21.8	72	5%	21	21	4	50	10%
	2020L1^g^	23.3	58	18%	35	22	19	6	23%
	2020L2	20.8	59	16%	28	24	18	15	23%
	2022	21.7	88	42% a^f^	29	12	7	10	50% a^f^
MUC14-228	2019	18.7	84	5%	53	14	4	25	7%
	2020L1	22.9	85	12%	58	10	10	10	15%
	2020L2	20.1	79	11%	56	12	13	7	14%
	2021	18.9	69	9%	27	38	14	13	12%
	2022	19.8	87	25% b	58	7	7	3	27%
Jal1	2020L1	42.0	5	0%	72	5	14	13	0%
	2020L2	58.0	3	0%	60	7	21	13	0%
	2021	47.1	9	0%	39	21	17	23	0%
	2022	na	0	2% e	60	6	24	9	2%
20G722-030	2022	26.1	98	19% bc	55	6	10	10	24% c
20G722-031	2022	23.0	95	35% a	39	11	5	11	41% b
20G722-032	2022	31.5	93	22% b	56	6	9	7	27% c
20G722-033	2022	32.5	100	14% cd	47	4	31	4	22% c
20G722-045	2022	21.9	85	9% d	66	9	7	8	11% d
Ds228MF76	2022	23.5	88	35% a	45	9	9	3	39% b

^a^ Destemmed is the fraction of mechanically harvested green marketable fruits perfectly destemmed.

^b^ Loose is the fraction of mechanically harvested green marketable fruits with stem attached.

^c^ Attached to branch is the fraction of green marketable fruits born on harvested branches.

^d^ Non-marketable is the fraction of mechanically harvested damaged, red, diseased and immature fruits.

^e^ Not harvested is the fraction of fruits remaining on plants or knocked to the ground and not recovered by the machine during mechanical harvest.

^f^ Destemmed fraction in 2022, values in the same column not connected by the same letter are significantly different based on comparisons of means using Student’s t (*p* < 0.05).

^g^ 2020L1 and 2020L2 indicates location 1 and location 2 at UC Davis in 2020.

### 3.3 Genetic control of easy-destemming

To understand the genetic regulation of mechanical harvest traits and implement marker-assisted selection, we genotyped 155 and 107 F_3_ families and parental lines from the MUC14 and GUC14 populations, respectively, using a modified genotype-by-sequencing (GBS) method. The MUC14 genetic map consisted of 1,402 genetic bins with a total length of 948 cM (Supplementary Data Sheet S5) and the GUC14 genetic map consisted of 847 genetic bins with a total length of 701 cM (Supplementary Data Sheet S7). Of the MUC14 families used for the genetic map, 151 were scored for destemming frequency and force using the torque watch in 2019. Heritability estimates from this data were high with broad sense heritability for destemming rate and force at 0.76 and 0.73, respectively, and narrow sense heritability at 0.54 and 0.61, respectively. QTL analysis was also carried out using destemming data collected in 2015 for all 107 GUC14 F_3_ families.

A combination of genome wide association analysis (GWAS), composite interval mapping and multiple QTL modeling applied to data collected in 2015, 2016 and 2019 identified nine genomic regions controlling destemming frequency and/or force. The QTL regions detected across years, populations, and methods were consistent (Table 2; Supplementary Table S2). Based on QTL models determined with the 2019 data, there were destemming frequency QTL on chromosomes 9 and 10 that explained 19% and 6% of the variation, respectively (Table 3). These regions overlapped with destemming force QTL. The chr10 QTL contributed up to 34% of the variation in destemming force and was observed in all trials (Table 2; Supplementary Table S2). Other force QTL generally had more moderate phenotypic effects with the exception of a QTL on chr6. However, this QTL was detected only in the 2016 trial. The estimated effects from the additive QTL model detected with the 2019 trial indicate semi-dominance to dominance, with the UCD-14 alleles contributing to higher destemming rate (positive effect) and lower destemming force (negative effect; Table 1). In addition to the QTL model from the 2019 trial, the destem force destem3.2 QTL was consistent with analyses using the GUC14 and MUC14 populations in 2015 while the destem4.2 QTL was observed for both force and destem rate in 2016 where a subset of the MUC14 population was phenotyped. Additional relatively minor QTL for destem rate were found on chromosomes 3 (dstem3.1) and 4 (dsem4.1), destem force on chromosome 2 (dstem2.1) and both rate and force on chromosome 11 (dstem11.1) that were environment and/or population specific.

**TABLE 2:** Summary of quantitative trait locus (QTL) detected for destemming force and destemming rate using IcImapping, and genome wide association studies (GWAS) with data collected from both GUC and MUC populations from 2015 to 2019.

QTL	Trait^a^	MUC14^b^	GUC14	GWAS	Chr	Left CI (bp)^c^	Right CI (bp)	% Var^d^	R/qtl model (2019)^e^	Confirmed with segregents^f^
dstem2.1	Force		X	X	2	140,431,201	end	13.9		X
dstem3.1	Rate		X		3	208,059,166	243,131,096	10.6—13.0		
dstem3.2	Force	X	X	X	3	244,579,706	end	8.1—14.4	X	X
dstem4.1	Rate	X			4	10,029,757	13,496,111	9.1—10.0		
dstem4.2	Rate	X			4	210,329,324	216,123,607	8.5—36.3		
	Force	X			4	196,923,359	207,768,277	5.6—16.6	X	
dstem6.1	Force	X			6	70,449,358	162,492,651	28.7—31.2		
dstem9.1	Rate	X			9	23,275,218	48,370,174	14.5—19.0	X	
	Force	X			9	43,674,098	61,354,220	7.0	X	
dstem10.1	Rate	X	X	X	10	138,256,069	204,089,386	5.9 - 23.2	X	
	Force	X			10	141,037,248	179,169,962	21.1—34.5	X	X
dstem11.1	Force	X			11	210,991,146	227,304,733	6.1—8.5	X	
	Rate			X	11	218,914,923				

^a^ Traits for which a QTL was identified, Force represents destemming force and Rate represents destemming rate.

^b^ The population and analysis that a QTL was identified including MUC14 population QTL, GUC14 population QTL, or GWAS with MUC14 and GUC14 populations combined.

^c^ The left and right confidence intervals based on all QTL analyses.

^d^ The percentage of phenotypic variance explained by QTL based on all analyses.

^e^ The QTL that were components of the QTL models identified from the 2019 field trial.

^f^ The QTL that were validated using segregating populations.

**TABLE 3:** Quantitative trait locus (QTL) models for destemming frequency and force from analysis based on 151 MUC14 F_3_ families evaluated in 2019.

Trait	Model			QTL							
								Effect est^c^		1.5 LOD interval^d^	
	Model^a^	LOD	%Var^b^	Chr	Pos (cM)	LOD	%Var	Additive	Dominance	Left (cM)	Right (cM)
Destem rate	y ∼ Q1 + Q2	9.2	24.4	9	35.2	7.4	19.0	0.05	0.19	23.2	37.1
				10	34.0	2.5	5.9	0.04	0.11	26.5	40.8
Destem force	y ∼ Q1 + Q2 + Q3 + Q4 + Q5	25.6	55.9	3	97.9	5.3	8.1	−1.72	−3.82	93.5	102.7
				4	43.0	3.7	5.6	−1.34	−2.86	10.5	51.3
				9	36.5	4.8	7.3	−1.80	−2.34	35.2	43.3
				10	25.2	12.2	21.1	−1.91	−6.23	23.6	26.5
				11	67.0	4.0	6.1	−1.03	−3.34	56.4	73.4

^a^ In the QTL model; Q1, Q2, Q3, etc. Indicates each QTL for a trait (e.g., Destem Rate has two QTL: Q1 and Q2).

^b^ %Var is the percentage of phenotypic variance explained by the QTL model or individual QTL.

^c^ Effect Est is the estimate effect of QTLs (+ values indicate higher values are associated with UCD14;—values indicate higher values are associated with Maor).

^d^ 1.5 LOD interval is the left and right positions when the LOD score is decreased by 1.5 from the peak LOD value.

In order to validate destemming force QTL, three MUC14 families segregating for QTL on chrs 2, 3 or 10 were genotyped and evaluated for destemming force. Plants homozygous for the UCD-14 allele on chrs 2 and 3 had significantly lower destemming force (*p* < 0.0001) than homozygous ‘Maor’ or heterozygous individuals (Figure 3D). The chr10 locus exhibited a semi-dominant and larger effect. Plants with the UCD-14 allele on chr10 had significantly lower destemming force that those that were heterozygous or homozygous Maor (*p* < 0.0001) and heterozygous plants had lower destemming force than homozygous Maor plants (*p* < 0.05). The QTL regions on chrs 3 and 10 were also found to segregate with destemming force in trials carried out at NMSU. These data were used to guide the selection of seed to advance for crosses and increase for larger trials.

### 3.4 Cytology and genomics

**FIGURE 4:**
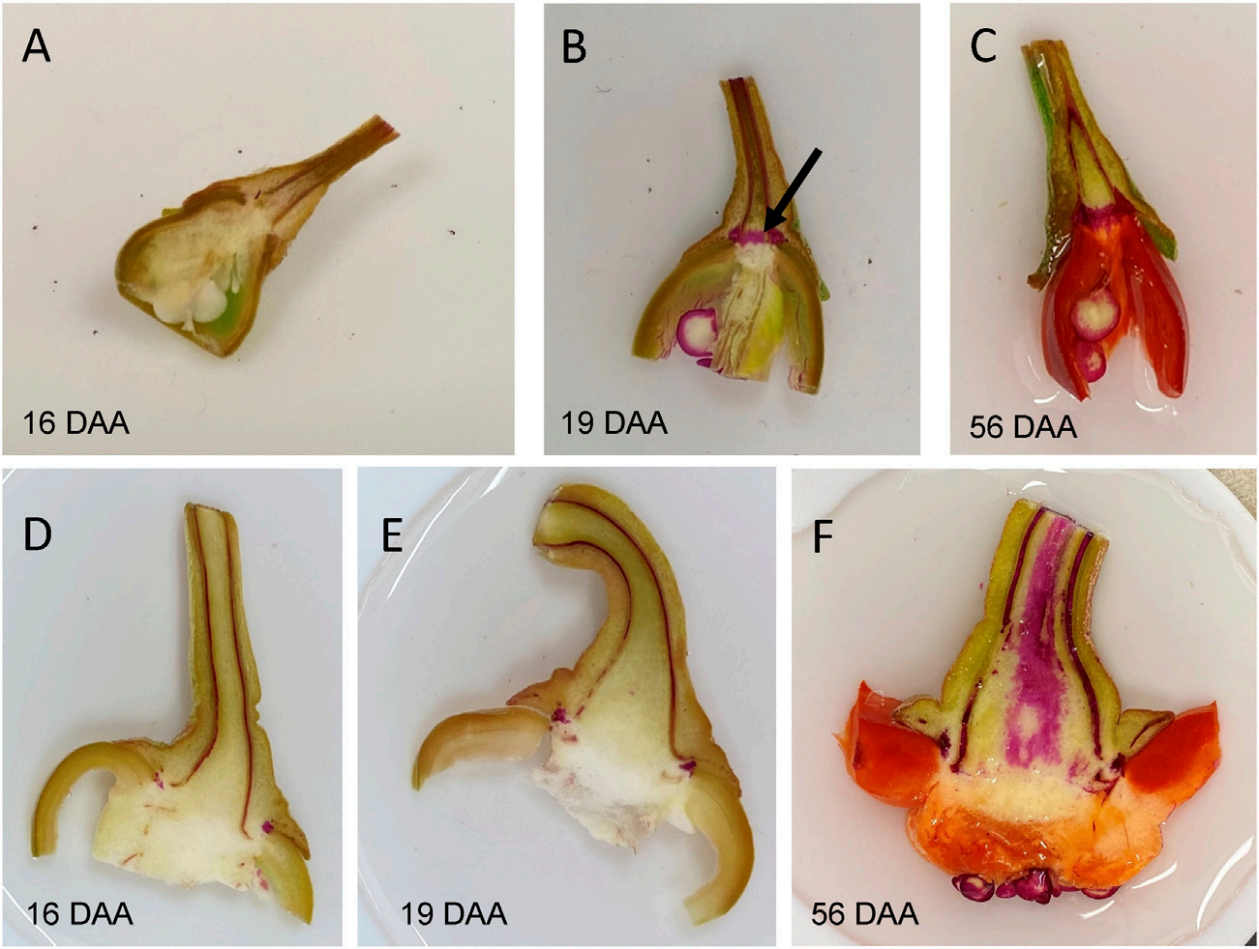
Phloroglucinol staining for lignin in fresh longitudinal cuts through the proximal end of fruit with pedicel of developing UCD14 **(A–C)** and Maor **(D–F)** fruits. **(B)** Lignification, indicated by red staining, of the region between the fruit and pedicel of UCD14 fruits (arrow) was first observed at 19 days after anthesis (DAA). **(F)** There was no sign of lignification between fruit and pedicel of the non-destemming line, Maor, even at the red ripe stage (56 DAA).

Preceding abscission, the tissues immediately distal to the abscission separation layer typically stain red with phloroglucinol-HCl treatment, an indicator of lignin deposition (Carns, 1966; Iwai et al., 2013; Mitra and Loqué, 2014). We observed staining appearing at the receptacle abscission zone of developing and mature green UCD-14 pepper fruits beginning at 19–26 days after anthesis while staining of the same region of non-destemming Maor fruits was absent, even at the red ripe stage (Figure 4). These results indicated that easy-destemming of mature green UCD-14 fruits is due to the presence and early activation of the pedicel/fruit abscission zone.

**FIGURE 5:**
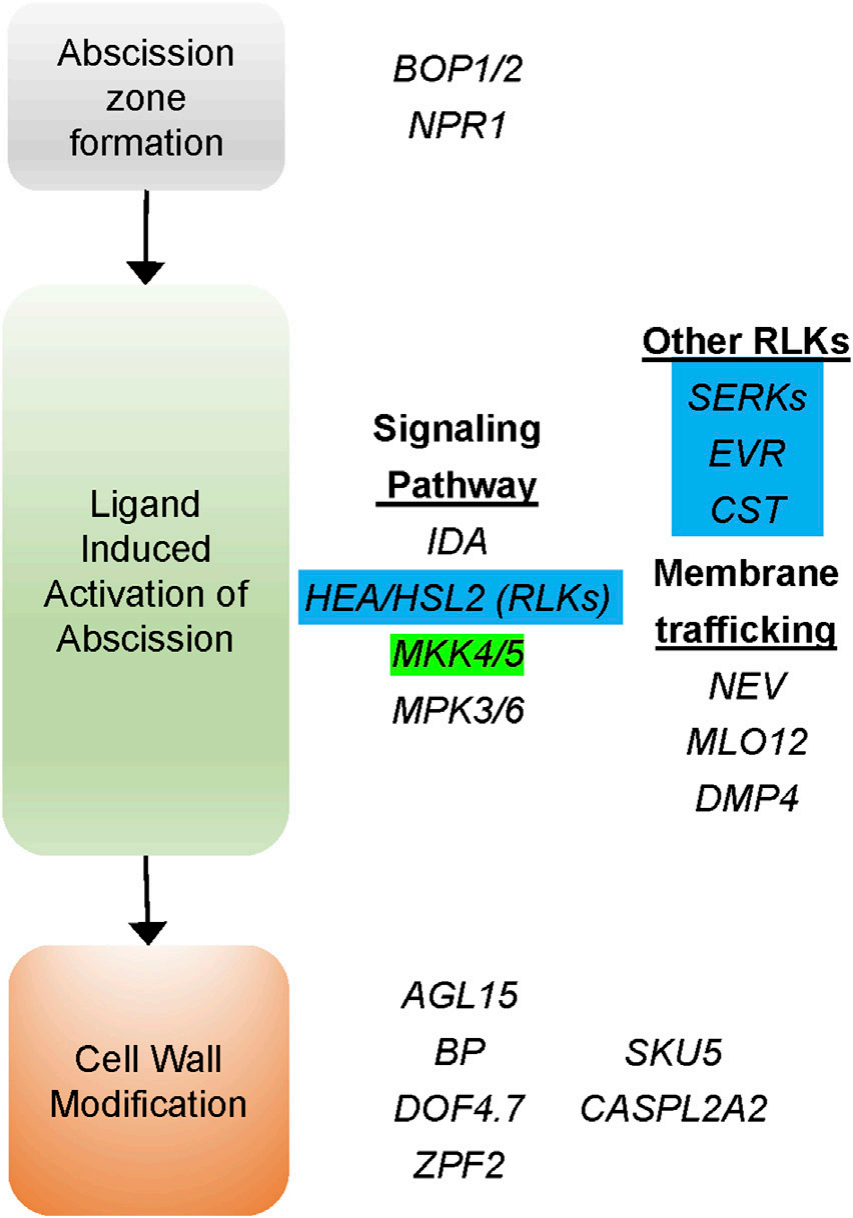
Genes affecting organ abscission based on *Arabidopsis* mutants that affect floral organ abscission. Several likekinases (RLKs) are involved in the signaling pathway promoting the activation of abscission induced by interaction with the IDA ligand. Pepper homologs encoding four of these proteins were identified within destemming QTL intervals (highlighted in blue). In addition, a gene encoding the pepper homolog of the signal transduction kinase MITOGEN-ACTIVATED PROTEIN KINASE KINASE5 (MKK5) (highlighted in green) was also found under the dstem3.2 QTL.

Genes that control abscission in *Arabidopsis* and tomato were used to identify *C. annuum* homologs using reciprocal searches with the basic local alignment search tool (BLAST) (Altschul et al., 1990) at NCBI and the Sol Genomics Network (SGN) (Fernandez-Pozo et al., 2015). A total of seven genes involved in organ abscission were found within the destem2.1, 3.2, 4.1, 4.2, and 9.1 QTL regions (Supplementary Table S3). Five of the seven genes are homologs of *Arabidopsis* genes involved in the receptor kinase signal transduction pathway that activates floral organ abscission (Jinn et al., 2000; Butenko et al., 2003). Four of these genes encode receptor-like kinases (RLKs) that interact at the plasma membrane, including HSL2 (dstem2.1), SERK1 (dstem4.2), EVR (dstem3.2) and CST (dstem4.1), to regulate abscission in *Arabidopsis* (Figure 5) (Cho et al., 2008; Leslie et al., 2010; Lewis et al., 2010; Burr et al., 2011; Liljegren, 2012; Meng et al., 2016). One gene encodes a MITOGEN-ACTIVATED PROTEIN KINASE KINASE5 (MKK5) that is involved in transducing the signal downstream of the membrane associated RLKs in *Arabidopsis* (Cho et al., 2008). These data suggest that changes in key components of the abscission signal transduction pathway may be responsible for the early activation of the pedicel/fruit abscission zone and that these genes may regulate easy-destemming in UCD14-derived lines of pepper.

### 3.5 Breeding for easy-destemming jalapeño

In order to integrate the destemming phenotype into jalapeño-type peppers, F_5_ individuals from three consistently low destemming lines, MUC14-200, -228 and -297 were selected to cross with several jalapeño hybrids to generate backcross (BC) populations. These individuals were genotyped with markers on chrs 2, 3, and 10 that were designed based on preliminary QTL analysis (2015 data). All were found to be homozygous UCD-14 at chr10. The MUC14-228 destemming parent selected for crosses had favorable alleles at all three QTL while the MUC14-200 and MUC14-297 plants lacked the favorable allele at chr 2 and 3, respectively. Crosses with MUC14-200 and -228 were selected to advance based on genotype and phenotype. Since QTL markers were based on Maor and UCD-14 sequence, ten jalapeño hybrids were sequenced to identify informative SNPs between UCD-14 and jalapeño-types (Supplementary Table S1). Across trials, the QTL analyses identified the UCD-14 allele on chr10 as having a consistent and large effect on destemming rate and force (Table 2; Supplementary Table S2), and the QTL model from the 2019 data showed chr9 also having an effect on both destemming rate and force (Table 3). Based on the chr10 QTL genotype, BC_1_ plants were selected for backcrossing and generating BC_1_S_1_ families. The destemming x jalapeño families were evaluated in 2020 field trials. Approximately 184 individuals from each of five F_2_ and seven BC_1_S_1_ families were genotyped with QTL markers on chr9 and/or chr10 and 90 individuals per population carrying UCD14 allele(s) were selected to evaluate. In 2021, an additional 1,100 plants from four BC_1_S_1_ and 715 plants from five BC_2_S_1_ populations carrying UCD-14 alleles at chr9 and chr10 QTL were evaluated in the field.

Based on destemming, fruit size and shape, and plant architecture one F_2_, three BC_1_S_1_ and 2 BC_2_S_1_ populations were selected to advance. Seed were collected from the top 20 to 30 individuals from each selected population plus 10 exceptional individuals from the remaining populations. Single plants from each of the selected individuals, 128 from the 2020 field and 53 from the 2021 field, were grown in the greenhouse and evaluated for destem force, fruit size and shape, and plant architecture. Ten F_3_, 56 BC_1_S_2_ and 20 BC_2_S_2_ were selected to advance to F_4_, BC_1_S_3_, and BC_2_S_3_, respectively, and evaluated in replicated field trials in 2021 and 2022. Despite correlations between fruit size and shape traits (Supplementary Figure S2), we developed lines with good jalapeño size having low destem force. However, the best destemming families had an obtuse shape at the pedicel attachment (IPGRI, 1995). Five selected BC_1_S_3_ and one selected F_4_ were tested for efficiency of destemming with mechanical harvest in 2022 (Table 1). Two selections, 20G722-031 and Ds228MF76, had a significantly greater fraction of harvested destemmed fruits per total yield (35%, *p* < 0.05) and per harvested marketable yield (41% and 39%, respectively, *p* < 0.05) than the destemming donor parent MUC14-228 with 25% of total yield and 27% of harvested marketable yield destemmed by the harvester.

## 4 Discussion

Mechanical harvesting in pepper for both chiles and sweet types is an essential goal for the sustainability of the industry, especially for processing types that command a lower price, due to increasing cost and decreasing availability of labor for picking and/or destemming by hand (Funk and Marshall, 2012). This has been largely accomplished for ripe pepper crops such as red New Mexico-types, but not for green chiles (Walker and Funk, 2014). As demonstrated at UC Davis for tomato in the 1970s, successful mechanical harvesting requires a systems approach to combine harvesting technologies with varieties that can tolerate the process and maintain yield and quality (Funk et al., 2011). Several traits are essential, including erect stems, uniform ripening and fruit distribution, a thick pericarp tolerant of bruising and breakage combined with easy-destemming of the fruit for processing. Here we present initial efforts toward enabling mechanical harvest of green chile without the pedicel by leveraging a pepper landrace with high destemming rate and low destemming force.

The processing tomato industry was revolutionized by the combination of genetic mutations affecting shoot architecture. These mutations resulted in the self-pruning phenotype, a determinate trait resulting from a mutation in the SELF PRUNNING (SP) gene, and the jointless phenotype, where the joint within the pedicel is absent, thus strengthening the pedicel for easier removal. (Rick, 1967; 1978; Zahara and Scheuerman, 1988; Ito and Nakano, 2015). The shoot architecture of pepper differs from that of tomato in that the sympodial units are less branched, having only a single flower in most pepper varieties (Lippman et al., 2008; Elitzur et al., 2009; Cohen et al., 2012; Park et al., 2014). The single pepper flower is born on a jointless pedicel with an abscission zone at the pedicel/shoot boundary that is activated under stress conditions or when there is a lack of pollination resulting in flower drop. The two key traits enabling mechanical harvest of tomato don’t apply to pepper due to differences in shoot architecture.

Previous work showed a harvester with an incline counter-rotating double open-helix worked well for harvesting green chiles with the pedicel attached (Funk and Walker, 2010). The action of this type of harvester on pepper fruits is similar to that when the force is applied with a torque gauge. Therefore, we used destemming rate and force measured with the torque gauge at the mature green stage as the criteria for selecting lines that are more likely to harvest well without the pedicel at the mature green stage. This approach was successful for selecting destemming lines from multiple populations that have improved machine harvest. When lines with a range of average destemming force were subjected to machine harvest, the amount of green destemmed fruits was correlated with destemming force.

Easy destemming in green chiles is different than fruit drop associated with the deciduous red-ripe fruits of wild *C. annuum* as easy-destemming fruit are mature but not ripe and do not drop naturally at the mature green or red-ripe stage. The dominant *S* locus in wild pepper confers the abscission of red-ripe fruit, thus easy removal of fruit without the pedicel during picking. However, a pleiotropic or tightly linked effect of undesirable pericarp softening has precluded its use for machine harvest (Paran and Van Der Knaap, 2007). Conversely, we show that we can introgress easy-destemming at the mature green stage conferred by the landrace UCD-14 while maintaining desirable pericarp firmness. In addition, UCD-14 fruits remain firm even at the red ripe stage. Although the destemming trait is highly heritable, it seems to be controlled by a major locus on pepper chr10, but modified by several other loci depending on genetic background and environment. We note that QTL identified do not overlap with the *S* locus (Hu et al., 2022). The gene associated with the *S* locus, Capana10g002229, is believed to encode a polygalacturonase, a cell wall degrading enzyme involved in fruit ripening (Fischer and Bennett, 1991; Rao and Paran, 2003; Hu et al., 2022). This gene is on chr10 at positions 217,699,094 to 217,705,135 in the UCD10Xv1.1 genome, more than 13 Mb beyond the dstem10.1 QTL.

Abscission zones become lignified prior to abscission. The presence of lignin at the pedicel/fruit boundary of UCD-14 was observed indicating the destemming trait may be related to the presence and activation of an abscission zone. This is similar to what has been observed for the citrus fruit abscission zone (AZ-C) and consistent with abscission of plant organs such as floral organs in *Arabidopsis* (Rajani and Sundaresan, 2001; Merelo et al., 2017). We detected activation of an abscission layer in easy-destemming genotypes prior to the mature green stage and not in non-destemming types, even at the red ripe stage. Interestingly, candidate genes under several QTL are homologous to known regulators of the organ abscission pathway, which isn’t yet fully defined (Lee, 2019; Shi et al., 2019).

Genes thought to regulate *Arabidopsis* floral organ abscission are shown in Figure 5. The abscission-signaling cascade is induced by the secreted peptide INFLORESCENCE DEFICIENT IN ABSCISSION (IDA). The IDA peptide is bound by two receptor-like protein kinases (RLK) HEASA and HEASA-LIKE2 (HEA**/**HSL2). The IDA ligand promotes heterodimerization of HEA/HSL2 with LRR-RLK proteins that are members of the SOMATIC EMBRYOGENESIS RECEPTOR KINASE (SERK) family (Meng et al., 2016; Santiago et al., 2016). The ligand receptor complex activates a MAP-kinase cascade, including MKK5**,** activating KNOX transcription factors that induce the expression of cell wall remodeling and cell wall degrading enzymes (Shi et al., 2011). Interactions between the RLKs may be mediated *via* endosomal trafficking which is regulated by NEVERSHED (NEV), an ADP-ribosylation factor-GPase activating factor (ARF-GAP) (Liljegren et al., 2009). This membrane trafficking is regulated by the interaction of RLKs CAST AWAY **(**CST) and EVERSHED (EVR), which are thought to act as an inhibitors of abscission through interactions with HEA, HSL2, and SERKs, regulating the timing and location of abscission (Leslie et al., 2010; Burr et al., 2011). In our analysis, we find four prominent RLK encoding genes that are components of the organ abscission pathway segregating with destemming force and frequency in pepper. These include the promoters of abscission, *HSL2* and *SERK1*, and putative negative regulators of abscission, *CST* and *EVR*.

The results of the destemming QTL analysis indicate UCD-14 alleles promote fruit abscission with additive QTL effects. In *Arabidopsis*, HSL2 and SERK1 functions promote organ abscission. The UCD-14 allele at the QTL on chr 2, where HSL2 was found, appears semidominant based on IciMapping and dominant based on the QTL validation study shown in Figure 2. The R/qtl model for destemming force indicates the UCD-14 allele at the QTL on chr4, where SERK1 was found, promotes abscission and is dominant. This would be consistent with the selection for reduced fruit abscission during domestication which has been observed for many species (Paran and Van Der Knaap, 2007; Li and Olsen, 2016). The functions of EVR and CST are less straightforward to interpret since *Arabidopsis evr* and *cst* mutants promote abscission in the non-abscising *nev* mutant background (Leslie et al., 2010; Burr et al., 2011). Therefore, EVR and CST are thought of as negative regulators of abscission. On their own, mutations in either EVR or CST have no effect on *Arabidopsis* floral organ shedding. Our results indicate UCD-14 alleles at the destem3.2 and destem4.1 QTL, where *EVR* and *CST* are found, promote abscission contributing to higher destemming rate and lower destemming force.

The SERK family proteins redundantly play a role in plant growth, development, immune response and abiotic stress response (Kaur et al., 2023). The MKKs also play a role in these processes (Osei-Wusu, 2022; Sun and Zhang, 2022). The functions of HSL2, EVR and CST appear more specific to organ abscission. We observed correlations between easy-destemming and fruit size, shape, and pericarp thickness, however it is unclear if these traits have a direct physiological role on destemming or are genetically linked to destemming. We did not observe any association between destemming and plant architecture or stress response although we have not done in-depth phenotyping for these traits. Populations derived from UCD-14 have reduced yield by weight, which is likely due to reduced fruit size. These traits are important for the development of a commercially viable jalapeno line that can be mechanically harvested and therefore are targets of our continued breeding efforts to improve fruit yield and quality.

## 5 Conclusion

We describe a novel phenotype that combines easy-destemming with acceptable pericarp quality at the mature green stage in pepper, critical to green chile industry including jalapeño, serrano, and New Mexico-type peppers. The genetic basis of the destemming trait seems quantitative with a major QTL on chr 10 and eight additional QTL having population and/or environment specific effects. The lignification of cells at the fruit/pedicel junction was observed during fruit development, consistent with the formation of an abscission zone in destemming genotypes. Candidate genes known to control floral organ abscission in *Arabidopsis* co-segregate with several QTL. We further show that we can introgress the trait into jalapeño-types and low destemming force enables mechanical harvesting of peppers. Easy-destemming is an essential trait that needs to be combined with plant architecture, yield, fruit quality, and management to fully achieve mechanical harvesting in pepper.

## Data Availability

The original contributions presented in the study are publicly available. This data can be found here: https://www.ebi.ac.uk/eva/?eva-study=PRJEB60339. Project: PRJEB60339. Analyses: ERZ16273090

## References

[B1] Abecasis G. R. Cardon L. R. Cookson W. (2000). A general test of association for quantitative traits in nuclear families. Am. J. Hum. Genet. 66 (1), 279–292. 10.1086/302698 10631157 PMC1288332

[B2] Altschul S. F. Gish W. Miller W. Myers E. W. Lipman D. J. (1990). Basic local alignment search tool. J. Mol. Biol. 215 (3), 403–410. 10.1016/S0022-2836(05)80360-2 2231712

[B84] Ashrafi H. Hill T. Stoffel K. Kozik A. Yao J. Chin-Wo S. R. (2012). De novo assembly of the pepper transcriptome (*Capsicum annuum*): A benchmark for in silico discovery of SNPs, SSRs and candidate genes. BMC Genomics 13 (1), 1–15. 23110314 10.1186/1471-2164-13-571 PMC3545863

[B81] Bolger A. M. Lohse M. Usadel B. (2014). Trimmomatic: A flexible trimmer for Illumina sequence data. Bioinformatics 30 (15), 2114–2120. 24695404 10.1093/bioinformatics/btu170 PMC4103590

[B3] Bosland P. W. Votava E. J. Votava E. M. (2012). Peppers: Vegetable and spice capsicums. Cambridge, MA: Cabi.

[B74] Bosland P. W. Walker S. (2014). Growing chiles in New Mexico (Guide H-230). Las Cruces: New Mexico State University Cooperative Extension Service. Available at: https://aces.nmsu.edu/pubs/_h/H230/welcome.html.

[B4] Bradbury P. J. Zhang Z. Kroon D. E. Casstevens T. M. Ramdoss Y. Buckler E. S. (2007). TASSEL: software for association mapping of complex traits in diverse samples. Bioinformatics 23 (19), 2633–2635. 10.1093/bioinformatics/btm308 17586829

[B5] Broman K. W. Wu H. Sen Ś. Churchill G. A. (2003). R/qtl: QTL mapping in experimental crosses. bioinformatics 19 (7), 889–890. 10.1093/bioinformatics/btg112 12724300

[B6] Burr C. A. Leslie M. E. Orlowski S. K. Chen I. Wright C. E. Daniels M. J. (2011). CAST AWAY, a membrane-associated receptor-like kinase, inhibits organ abscission in Arabidopsis. Plant Physiol. 156 (4), 1837–1850. 10.1104/pp.111.175224 21628627 PMC3149937

[B7] Butenko M. A. Patterson S. E. Grini P. E. Stenvik G.-E. Amundsen S. S. Mandal A. (2003). Inflorescence deficient in abscission controls floral organ abscission in Arabidopsis and identifies a novel family of putative ligands in plants. Plant Cell 15 (10), 2296–2307. 10.1105/tpc.014365 12972671 PMC197296

[B8] Carns H. R. (1966). Abscission and its control. Annu. Rev. Plant Physiol. 17, 295–314. 10.1146/annurev.pp.17.060166.001455

[B9] Chaim A. B. Paran I. Grube R. Jahn M. Van Wijk R. Peleman J. (2001). QTL mapping of fruit-related traits in pepper (*Capsicum annuum*). Theor. Appl. Genet. 102 (6), 1016–1028. 10.1007/s001220000461

[B10] Cho S. K. Larue C. T. Chevalier D. Wang H. Jinn T.-L. Zhang S. (2008). Regulation of floral organ abscission in *Arabidopsis thaliana* . Proc. Natl. Acad. Sci. 105 (40), 15629–15634. 10.1073/pnas.0805539105 18809915 PMC2563077

[B11] Chunthawodtiporn J. Hill T. Stoffel K. Van Deynze A. (2018). Quantitative Trait loci controlling fruit size and other horticultural Traits in bell pepper (*Capsicum annuum*). Plant Genome 11 (1), 160125. 10.3835/plantgenome2016.12.0125 PMC12899126 29505638

[B12] Cohen O. Borovsky Y. David-Schwartz R. Paran I. (2012). CaJOINTLESS is a MADS-box gene involved in suppression of vegetative growth in all shoot meristems in pepper. J. Exp. Bot. 63 (13), 4947–4957. 10.1093/jxb/ers172 22859675 PMC3427992

[B13] Covarrubias-Pazaran G. (2016). Genome-assisted prediction of quantitative traits using the R package sommer. PloS one 11 (6), e0156744. 10.1371/journal.pone.0156744 27271781 PMC4894563

[B14] Covarrubias-Pazaran G. (2018). Quantitative genetics using the sommer package. Vienna: R Found. Stat. Comput. Available at: https://cran.r-project.org/web/packages/sommer/vignettes/sommer.pdf (*accessed* *Mar* *22*, *2018*).

[B15] Elitzur T. Nahum H. Borovsky Y. Pekker I. Eshed Y. Paran I. (2009). Co-Ordinated regulation of flowering time, plant architecture and growth by FASCICULATE: the pepper orthologue of SELF PRUNING. J. Exp. Bot. 60 (3), 869–880. 10.1093/jxb/ern334 19174461 PMC2652051

[B16] Fernandez-Pozo N. Menda N. Edwards J. D. Saha S. Tecle I. Y. Strickler S. R. (2015). The Sol genomics Network (SGN)—from genotype to phenotype to breeding. Nucleic acids Res. 43 (D1), D1036–D1041. 10.1093/nar/gku1195 25428362 PMC4383978

[B17] Fischer R. L. Bennett A. B. (1991). Role of cell wall hydrolases in fruit ripening. Annu. Rev. plant Biol. 42 (1), 675–703. 10.1146/annurev.pp.42.060191.003331

[B18] Funk P. A. Marshall D. E. (2012). “1 7 pepper harvest technology,” in Peppers: Botany, production and uses, 227.

[B19] Funk P. A. Walker S. J. (2010). Evaluation of five green Chile cultivars utilizing five different harvest mechanisms. Appl. Eng. Agric. 26 (6), 955–964. 10.13031/2013.35906

[B20] Funk P. A. Walker S. J. Herbon R. P. (2011). A systems approach to Chile harvest mechanization. Int. J. Veg. Sci. 17 (3), 296–309. 10.1080/19315260.2010.549167

[B21] Ganguly S. Praveen P. K. Para P. A. Sharma V. (2017). Medicinal properties of chilli pepper in human diet: an editorial. ARC J. Public Health Commun. Med. 2 (1), 6–7.

[B22] Gonda I. Ashrafi H. Lyon D. A. Strickler S. R. Hulse-Kemp A. M. Ma Q. Y. (2019). Sequencing-based bin map construction of a Tomato mapping population, facilitating high-resolution quantitative Trait loci detection. Plant Genome 12 (1), 180010. 10.3835/plantgenome2018.02.0010 PMC12809921 30951101

[B78] Havlik C. D. Walker S. J. Funk P. Marsalis M. A. (2018). “Optimum plant spacing for New Mexico green chile (Capsicum annuum) mechanical harvest efficiency,” in 2018 ASHS Annual Conference (ASHS).

[B23] Herbon R. P. Cillessen D. E. Gamillo E. M. Hyde A. M. (2009). “Engineering a machine to remove stems from Chile peppers-a critical need for the new Mexico Chile industry,” in 2009 Reno, Nevada, June 21-June 24, 2009 (American Society of Agricultural and Biological Engineers).

[B24] Howard L. R. Talcott S. T. Brenes C. H. Villalon B. (2000). Changes in phytochemical and antioxidant activity of selected pepper cultivars (Capsicum species) as influenced by maturity. J. Agric. Food Chem. 48 (5), 1713–1720. 10.1021/jf990916t 10820084

[B25] Hu F. Dong J. Zhang S. Song Z. Guan W. Yuan F. (2022). Fine mapping and gene silencing pinpoint Capana10g002229 as a strong candidate gene regulating the deciduous character of ripe pepper fruit (Capsicum spp.). Res. Sq. [Preprint]. 10.21203/rs.3.rs-1901496/v1 37037971

[B82] Hulse-Kemp A. M. Maheshwari S. Stoffel K. Hill T. A. Jaffe D. Williams S. R. (2018). Reference quality assembly of the 3.5-Gb genome of Capsicum annuum from a single linked-read library. Hortic. Res. 5. 10.1038/s41438-017-0011-0 PMC5798813 29423234

[B26] IBISWORLD (2021). Hot sauce production in the US: Market research report. [Online]. Available: https://www.ibisworld.com/united-states/market-research-reports/hot-sauce-production-industry/ (Accessed October 2022).

[B27] IPGRI (1995). CATIE. Descriptors for Capsicum (Capsicum spp.), 110. Rome, Italy: International Plant Genetic Resources Institute. the Asian Vegetable Research and Development Center, Taipei, Taiwan, and the Centro Agronómico Tropical de Investigación y Enseñanza. Turrialba, Costa Rica.

[B28] Ito Y. Nakano T. (2015). Development and regulation of pedicel abscission in tomato. Front. Plant Sci. 6, 442. 10.3389/fpls.2015.00442 26124769 PMC4462994

[B29] Iwai H. Terao A. Satoh S. (2013). Changes in distribution of cell wall polysaccharides in floral and fruit abscission zones during fruit development in tomato (*Solanum lycopersicum*). J. Plant Res. 126 (3), 427–437. 10.1007/s10265-012-0536-0 23124772

[B30] Jinn T.-L. Stone J. M. Walker J. C. (2000). HAESA, an Arabidopsis leucine-rich repeat receptor kinase, controls floral organ abscission. Genes Dev. 14 (1), 108–117. 10.1101/gad.14.1.108 10640280 PMC316334

[B31] Kaur A. Sharma A. Upadhyay S. K. (2023). “Role of somatic embryogenesis receptor-like kinase family in plants,” in Plant receptor-like kinases (Elsevier), 121–138.

[B32] Kraft K. Jesús Luna-Ruíz J. Gepts P. (2013). A new collection of wild populations of Capsicum in Mexico and the southern United States. Genet. Resour. Crop Evol. 60 (1), 225–232. 10.1007/s10722-012-9827-5

[B83] Langmead B. Salzberg S. L. (2012). Fast gapped-read alignment with Bowtie 2. Nat. Methods 9 (4), 357–359. 22388286 10.1038/nmeth.1923 PMC3322381

[B33] Lee Y. (2019). More than cell wall hydrolysis: Orchestration of cellular dynamics for organ separation. Curr. Opin. plant Biol. 51, 37–43. 10.1016/j.pbi.2019.03.009 31030063

[B34] Leslie M. E. Lewis M. W. Youn J.-Y. Daniels M. J. Liljegren S. J. (2010). The EVERSHED receptor-like kinase modulates floral organ shedding in Arabidopsis. Development 137 (3), 467–476. 10.1242/dev.041335 20081191 PMC2858908

[B35] Lewis M. W. Leslie M. E. Fulcher E. H. Darnielle L. Healy P. N. Youn J. Y. (2010). The SERK1 receptor‐like kinase regulates organ separation in Arabidopsis flowers. Plant J. 62 (5), 817–828. 10.1111/j.1365-313X.2010.04194.x 20230490 PMC2884084

[B36] Li L.-F. Olsen K. (2016). To have and to hold: selection for seed and fruit retention during crop domestication. Curr. Top. Dev. Biol. 119, 63–109. 10.1016/bs.ctdb.2016.02.002 27282024

[B37] Liljegren S. J. Leslie M. E. Darnielle L. Lewis M. W. Taylor S. M. Luo R. (2009). Regulation of membrane trafficking and organ separation by the NEVERSHED ARF-GAP protein. Development 136 (11), 1909–1918. 10.1242/dev.033605 19429787 PMC2680113

[B38] Liljegren S. J. (2012). Organ abscission: exit strategies require signals and moving traffic. Curr. Opin. plant Biol. 15 (6), 670–676. 10.1016/j.pbi.2012.09.012 23047135

[B39] Lippman Z. B. Cohen O. Alvarez J. P. Abu-Abied M. Pekker I. Paran I. (2008). The making of a compound inflorescence in tomato and related nightshades. PLoS Biol. 6 (11), e288. 10.1371/journal.pbio.0060288 19018664 PMC2586368

[B40] Marshall D. Boese B. (1998). Breeding Capsicum for mechanical harvest. Part2-Equipment. Proc. 10, 61–64.

[B41] Marshall D. (1995). “Mechanical pepper harvesting,” in I international symposium on solanacea for fresh market, 285–292.()

[B42] Marshall D. E. (1997). Designing a pepper for mechanical harvest. Capsicum Eggplant Nwsl. 16, 15–27.

[B43] Meng L. Li H. Zhang L. Wang J. (2015). QTL IciMapping: integrated software for genetic linkage map construction and quantitative trait locus mapping in biparental populations. Crop J. 3 (3), 269–283. 10.1016/j.cj.2015.01.001

[B44] Meng X. Zhou J. Tang J. Li B. de Oliveira M. V. Chai J. (2016). Ligand-induced receptor-like kinase complex regulates floral organ abscission in Arabidopsis. Cell Rep. 14 (6), 1330–1338. 10.1016/j.celrep.2016.01.023 26854226 PMC4758877

[B45] Merelo P. Agustí J. Arbona V. Costa M. L. Estornell L. H. Gómez-Cadenas A. (2017). Cell wall remodeling in abscission zone cells during ethylene-promoted fruit abscission in citrus. Front. plant Sci. 8, 126. 10.3389/fpls.2017.00126 28228766 PMC5296326

[B46] Miles J. Hinz W. Pike W. (1978). Development of a mechanism for picking Chile peppers. Trans. ASAE 21, 419–421.

[B47] Mitra P. P. Loqué D. (2014). Histochemical staining of *Arabidopsis thaliana* secondary cell wall elements. JoVE J. Vis. Exp. (87), e51381. 10.3791/51381 PMC4186213 24894795

[B80] Monson-Miller J. Sanchez-Mendez D. C. Fass J. Henry I. M. Tai T. H. Comai L. (2012). Reference genome-independent assessment of mutation density using restriction enzyme-phased sequencing. BMC genomics 13 (1), 1–15. 22333298 10.1186/1471-2164-13-72 PMC3305632

[B48] Osei-Wusu M. O. (2022). The role of Arabidopsis MAP kinase genes in biotic and abiotic stress signalling. [Doctoral dissertation]. Edinburgh, Scotland: Heriot-Watt University.

[B49] Palevitch D. Levy A. (1984). Horticultural aspects of mechanized sweet pepper harvesting. Amman, Jordan: Arab Scientific Conference of Biological Sciences.

[B50] Paran I. Van Der Knaap E. (2007). Genetic and molecular regulation of fruit and plant domestication traits in tomato and pepper. J. Exp. Bot. 58 (14), 3841–3852. 10.1093/jxb/erm257 18037678

[B51] Park S. J. Eshed Y. Lippman Z. B. (2014). Meristem maturation and inflorescence architecture—Lessons from the solanaceae. Curr. Opin. Plant Biol. 17, 70–77. 10.1016/j.pbi.2013.11.006 24507497

[B52] Pawar S. Bharude N. Sonone S. Deshmukh R. Raut A. Umarkar A. (2011). Chillies as food, spice and medicine: a perspective. Int. J. Pharm. Biol. Sci. 1 (3), 311–318.

[B53] Rajani S. Sundaresan V. (2001). The Arabidopsis myc/bHLH gene ALCATRAZ enables cell separation in fruit dehiscence. Curr. Biol. 11 (24), 1914–1922. 10.1016/s0960-9822(01)00593-0 11747817

[B54] Rao G. Paran I. (2003). Polygalacturonase: a candidate gene for the soft flesh and deciduous fruit mutation in Capsicum. Plant Mol. Biol. 51 (1), 135–141. 10.1023/a:1020771906524 12602897

[B55] Rick C. M. (1967). Fruit and pedicel characters derived from Galápagos Tomatoes. Econ. Bot. 21 (2), 171–184. 10.1007/bf02897867

[B56] Rick C. M. (1978). The tomato. Sci. Am. 239 (2), 76–87. 10.1038/scientificamerican0878-76

[B57] RStudio Team (2020). RStudio: integrated development for R. PBC. Boston, MA: Rstudio Team. URL http://www.rstudio.com .

[B58] Saleh B. Omer A. Teweldemedhin B. (2018). Medicinal uses and health benefits of chili pepper (Capsicum spp.): a review. MOJ Food Process Technol. 6 (4), 325–328. 10.15406/mojfpt.2018.06.00183

[B59] Santiago J. Brandt B. Wildhagen M. Hohmann U. Hothorn L. A. Butenko M. A. (2016). Mechanistic insight into a peptide hormone signaling complex mediating floral organ abscission. Elife 5, e15075. 10.7554/eLife.15075 27058169 PMC4848090

[B60] Shi C.-L. Stenvik G.-E. Vie A. K. Bones A. M. Pautot V. Proveniers M. (2011). Arabidopsis class I KNOTTED-like homeobox proteins act downstream in the IDA-HAE/HSL2 floral abscission signaling pathway. Plant Cell 23 (7), 2553–2567. 10.1105/tpc.111.084608 21742991 PMC3226213

[B61] Shi C.-L. Alling R. M. Hammerstad M. Aalen R. B. (2019). Control of organ abscission and other cell separation processes by evolutionary conserved peptide signaling. Plants 8 (7), 225. 10.3390/plants8070225 31311120 PMC6681299

[B62] Shooter S. B. Buffinton K. W. (1999). “Design and development of the pik rite chili pepper harvester: a collaborative project with the University, industry, and government,” in FIE'99 Frontiers in Education. 29th Annual Frontiers in Education Conference. Designing the Future of Science and Engineering Education. Conference Proceedings (IEEE Cat. No. 99CH37011) (IEEE). 12B14/19-12B14/24.

[B63] Sun T. Zhang Y. (2022). MAP kinase cascades in plant development and immune signaling. EMBO Rep. 23 (2), e53817. 10.15252/embr.202153817 35041234 PMC8811656

[B64] Takele E. Daugovish O. Vue M. (2013). Costs and profitability analysis for bell pepper production in the Oxnard Plain. Ventura County: University of California ANR, 2012–2013. Fresh bell pepper production.

[B65] Walker S. J. Funk P. A. (2014). Mechanizing Chile peppers: Challenges and advances in transitioning harvest of new Mexico’s signature crop. HortTechnology 24 (3), 281–284. 10.21273/horttech.24.3.281

[B66] Walker S. Wall M. M. Bosland P. W. (2004). `NuMex Garnet' paprika. HortScience 39 (3), 629–630. 10.21273/hortsci.39.3.629

[B67] Walker S. J. Funk P. Joukhadar I. Place T. Havlik C. Tonnessen B. (2021). ‘NuMex odyssey’, a new Mexico–type green Chile pepper for mechanical harvest. HortScience 56 (12), 1605–1607. 10.21273/hortsci15793-21

[B68] Wu Y. Bhat P. Close T. J. Lonardi S. (2007). “Efficient and accurate construction of genetic linkage maps from noisy and missing genotyping data,” in International workshop on algorithms in bioinformatics (Springer), 395–406.

[B69] Wu Y. Bhat P. R. Close T. J. Lonardi S. (2008). Efficient and accurate construction of genetic linkage maps from the minimum spanning tree of a graph. PLoS Genet. 4 (10), e1000212. 10.1371/journal.pgen.1000212 18846212 PMC2556103

[B79] Xin Z. Chen J. (2012). A high throughput DNA extraction method with high yield and quality. Plant Methods 8 (1), 1–7. 22839646 10.1186/1746-4811-8-26 PMC3441248

[B70] Yin L. Zhang H. Tang Z. Xu J. Yin D. Zhang Z. (2021). rMVP: a memory-efficient, visualization-enhanced, and parallel-accelerated tool for genome-wide association study. Genomics, Proteomics Bioinforma. 19 (4), 619–628. 10.1016/j.gpb.2020.10.007 PMC9040015 33662620

[B71] Yu J. Pressoir G. Briggs W. H. Vroh Bi I. Yamasaki M. Doebley J. F. (2006). A unified mixed-model method for association mapping that accounts for multiple levels of relatedness. Nat. Genet. 38 (2), 203–208. 10.1038/ng1702 16380716

[B72] Zahara M. Scheuerman R. (1988). Hand-harvesting jointless vs. jointed-stem tomatoes. Calif. Agric. 42 (3), 14.

[B73] Zhang D. Choi D. W. Wanamaker S. Fenton R. D. Chin A. Malatrasi M. (2004). Construction and evaluation of cDNA libraries for large-scale expressed sequence tag sequencing in wheat (*Triticum aestivum* L.). Genetics 168 (2), 595–608. 10.1534/genetics.104.034785 15514038 PMC1448820

