# Supplementary material for: Genetics of destemming in pepper: A step towards mechanical harvesting
Source: Front Genet. 2023 Mar 17;14:1114832. doi: 10.3389/fgene.2023.1114832 (PMC10064014; doi:10.3389/fgene.2023.1114832)
Supplement: Supplementary file 5 [file Presentation1.pdf]

Supplementary Figures

A

p \ r	2015 Pull Rate	2016 Pull Rate	2016 Torque Rate	2015Pull Force	2016 Pull Force	2016 Torque Force
2015 Pull Rate	1	0.68	0.51	-0.52	-0.62	-0.47
2016 Pull Rate	0.0036	1	0.79	-0.73	-0.60	-0.68
2016 Torque Rate	0.0890	0.0025	1	-0.75	-0.64	-0.74
2015Pull Force	0.0398	0.0013	0.0052	1	0.70	0.67
2016 Pull Force	0.0110	0.0131	0.0249	0.0028	1	0.66
2016 Torque Force	0.1189	0.0149	0.0061	0.0173	0.0200	1

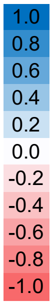

B

p \ r	2016 Rate	2019 Rate	2016 Force	2019 Force
2016 Rate	1	0.57	-0.20	-0.42
2019 Rate	0.0001	1	0.05	-0.32
2016 Force	0.2252	0.7537	1	0.48
2019 Force	0.0096	0.0511	0.0026	1

C

p \ r	2019 Destem Rate	2020 Rate	2019 Destem Force	2020 Force
2019 Destem Rate	1	0.63	0.31	0.25
2020 Rate	0.0149	1	0.24	0.33
2019 Destem Force	0.276	0.3987	1	0.90
2020 Force	0.39	0.2478	<.0001	1

Supplemental Figure 1. Correlations of destemming measurements with values in upper right triangles representing Pearson correlations and corresponding p values shown in the lower left. A) Correlations between 15 F3 families grown in both 2015 and 2016 for destem rate and destem force using pull force gauge and torque gauge. B) Destem force and rate correlations between 40 F3 families grown in both 2016 and 2019. C) Correlations between destem force and rate data collected in 2019 and 2020 from 13 F4 to F6 MUC14 RILs derived from F3 families selected for low destem force and high destem rate.

r / p	Destem Rate	Destem Force	Paricarp Thickness	Locule Number	Pedicel End Shape	Blossom End Shape	Fruit Length	Fruit Width	Ten Fruit Weight
Destem Rate	1	-0.288	-0.061	0.160	-0.231	0.023	0.132	-0.075	0.065
Destem Force	0.0005	1	0.426	0.073	0.472	0.202	0.140	0.534	0.357
Paricarp Thickness	0.4947	<.0001	1	0.199	0.545	0.179	0.307	0.694	0.677
Locule Number	0.0728	0.4125	0.0248	1	0.217	0.531	0.127	0.331	0.278
Pedicel End Shape	0.0085	<.0001	<.0001	0.0144	1	0.454	-0.058	0.669	0.490
Blossom End Shape	0.7949	0.0214	0.0435	<.0001	<.0001	1	-0.076	0.453	0.283
Fruit Length	0.1397	0.1187	0.0005	0.1567	0.5175	0.3996	1	0.263	0.489
Fruit Width	0.4069	<.0001	<.0001	0.0002	<.0001	<.0001	0.0029	1	0.766
Ten Fruit Weight	0.4625	<.0001	<.0001	0.0016	<.0001	0.0012	<.0001	<.0001	1

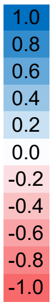

Supplemental Figure 2. Correlations between destemming measurements and fruit size and shape. Values in upper right triangles represent Pearson correlations with corresponding p values shown in the lower left.
